# Supplementary material for: Structural Impact of Selected Retinoids on Model Photoreceptor Membranes
Source: Membranes (Basel). 2023 Jun 1;13(6):575. doi: 10.3390/membranes13060575 (PMC10303579; doi:10.3390/membranes13060575)
Supplement: Supplementary file 1 [file membranes-13-00575-s001.zip › membranes-2279976-supplementary.pdf]

Membranes- 2279976

Title: Structural impact of selected retinoids on model photoreceptor membranes

Supplementary material

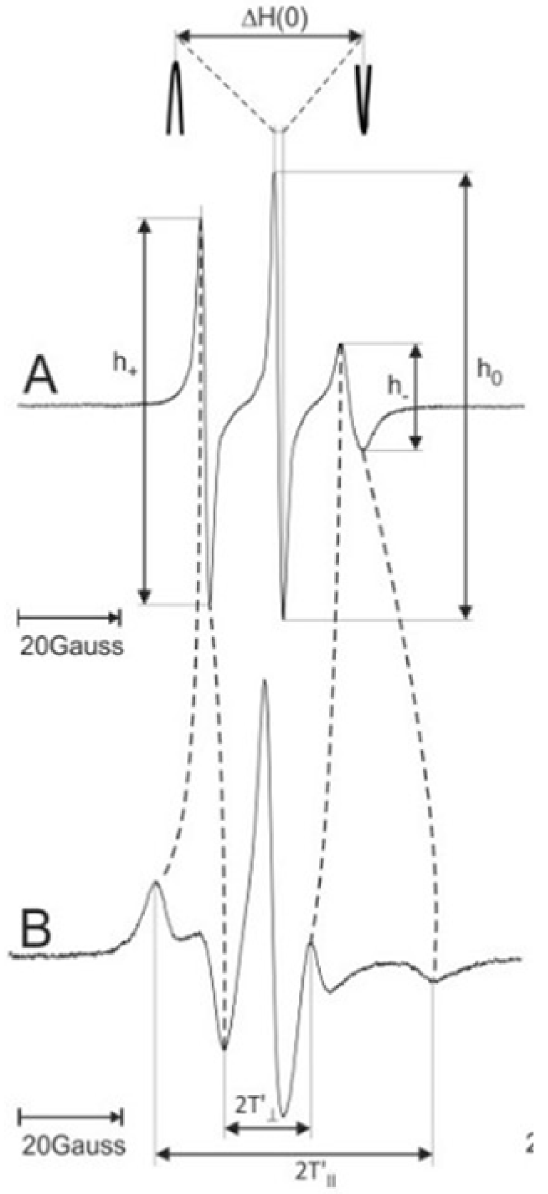

Figure S1 Representative spectra of 16-PC (A) and 5-PC (B) with marked key values for calculation of structural parameters (Marsh, D., *Electron Spin Resonance: Spin Labels*, in *Membrane Spectroscopy*, E. Grell, Editor. 1981, Springer Berlin Heidelberg: Berlin, Heidelberg. p. 51-142.; Berliner, L.J., *Spin labeling in enzymology: Spin-labeled enzymes and proteins*, in *Methods in Enzymology*. 1978, Academic Press. p. 418-480).

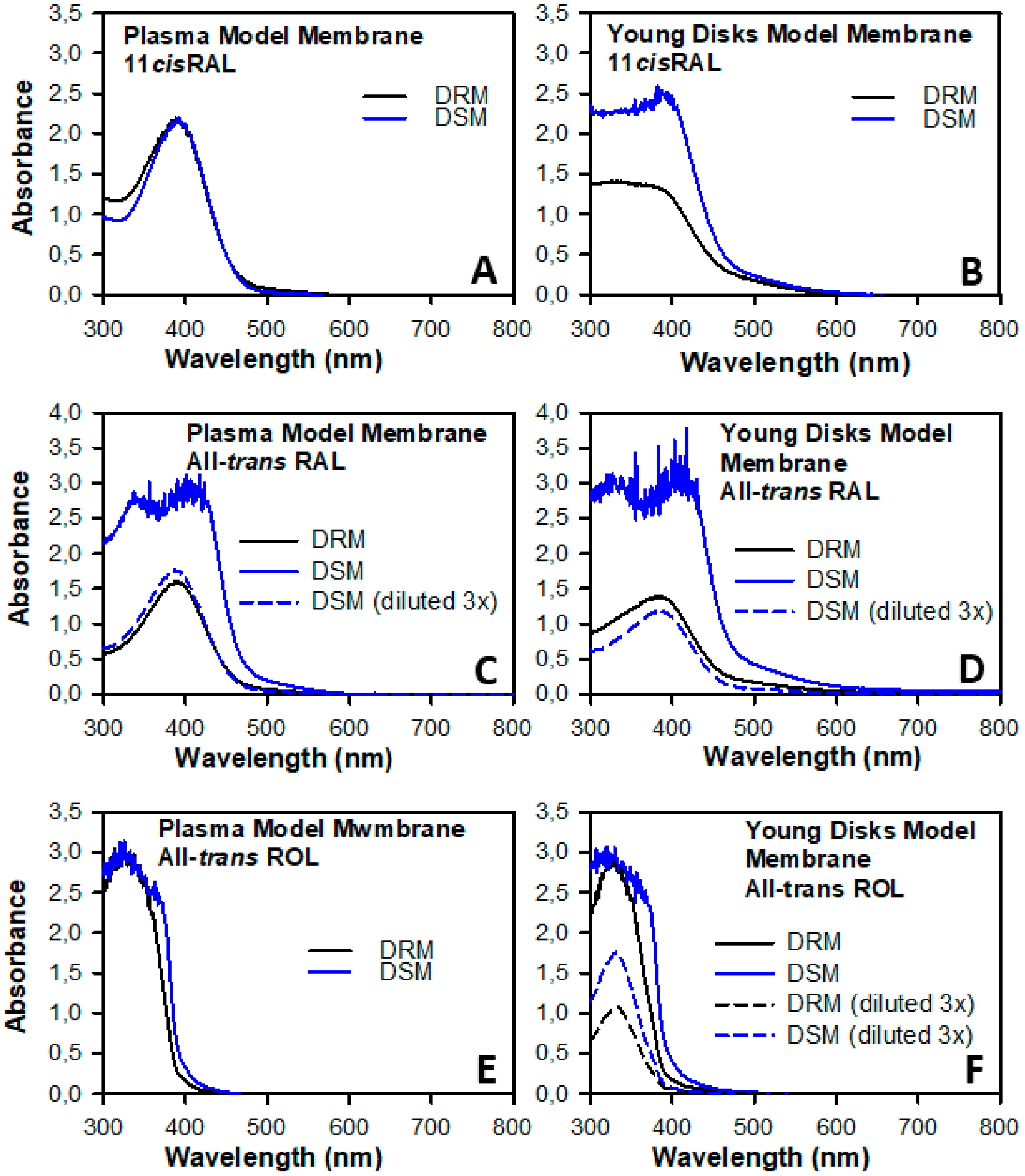

Figure S2 Absorption spectra of Folch's extracts of lipid domains: detergent resistant (DRM) and detergent sensitive (DSM) membrane domains formed in liposomes modelling photoreceptor plasma membrane and young discs membrane containing selected retinoids.

Separation of DRM and DSM has been performed according to the method previously described (Wiśniewska A and Subczyński WK, 2006, *Free Rad. Biol. Med*, **40:** 1820–1826; Gandhavadi M et al, 2002, *Biophys. J.*, **82:** 1469-1482; Ahmed SN et al., 1997, *Biochemistry*, **36:** 10944-10953). Shortly: liposomes composed of synthetic lipids of nature and ratio resembling lipid composition of photoreceptor plasma and young discs membranes (Table 1) containing selected retinoids (11cRAL, AtRAL and AtROL)

have been cooled down on ice and mixed with cold solution of Triton X-100 (1% w/v) in ratio 1:1. Mixtures of liposomes and detergent were incubated on ice for 30 min, then centrifuged (16 000 rcf, 30 min) at 4°C (bench Eppendorf Centrifuge 5424R). Pellets containing components of DRM domains have been carefully resuspended in 250 µl of fresh PBS buffer (20 mM, pH 7.4) while 250 µl of supernatants containing components of DSM domains have been carefully transferred to clean Eppendorf tubes. Hydrophobic components (including retinoids) present in supernatants and pellets have been extracted using a slightly modified Folch's method (Folch J. et al, 1957, J Biol Chem, 226(1): 497-509). Supernatant and resuspended pellet have been combined with Folch's extraction mixture (chloroform: methanol, 2:1, v/v) in 5:8 (v/v) ratio) and then vortexed vigorously for a few minutes. Samples were centrifuged (15000 rpm, 5 min, at 15°C) and the chloroform layer (containing retinoids) of each sample was collected. Absorption spectra of all samples have been acquired and presented in Figure S2.

Table S1 *Values of 2Azz in plasma membrane model in the absence and in the presence of selected retinoids*

**Plasma membrane**	**2Azz (G)**			
	**Control**	**+ 11cisRAL**	**+AtRAL**	**+AtROL**
T-PC	72,83±0,14	71,89±0,08 *	72,48±0,07	72,34±0,42
5-DOXYL	70,92±0,07	69,75±0,08 **	70,48±0,55	69,40±0,28 *
10-DOXYL	65,69±0,07	65,09±0,00 **	65,49±0,28	65,20±0,00 *
16-DOXYL	65,01±0,00	65,14±0,08	65,25±0,07 *	65,21±0,15

Table. S2 *Values of 2Azz in young disc membrane model in the absence and in the presence of selected retinoids*

**Young discs membrane model**	**2Azz (G)**			
	**Control**	**+ 11cisRAL**	**+AtRAL**	**+AtROL**
T-PC	72,63±0,14	71,89±0,40	71,89±0,24	72,90±0,24
5-DOXYL	68,82±0,00	68,91±0,32	68,94±0,44	69,19±0,08 *
10-DOXYL	65,25±0,07	65,37±0,08	65,20±0,00	65,76±0,16
16-DOXYL	65,30±0,00	65,54±0,16	65,20±0,00	65,31±0,32

Table S3 *Values of 2Azz in old disc membrane model in the absence and in the presence of selected retinoids*

**Old discs membrane model**	**2Azz (G)**			
	**Control**	**+ 11cisRAL**	**+AtRAL**	**+AtROL**
T-PC	71,65±0,69	70,71±0,40	73,21±0,27	72,11±0,40
5-DOXYL	67,74±0,00	68,12±0,32	67,67±0,17	68,29±0,08 *
10-DOXYL	66,28±0,14	66,17±0,08	66,09±0,01	66,38±0,08
16-DOXYL	65,25±0,21	66,04±0,72	65,46±0,05	65,14±0,24

***Table S4** Values of 2Azz in old disc membrane model in the absence and in the presence of all-trans retinal dimer*

**Old discs membrane**	**2Azz (G)**	
	**Control**	**+ AtRAL Dimer**
T-PC	71,65±0,69	71,55±0,87
5-DOXYL	67,74±0,00	69,30±0,56
7-DOXYL	-	68,18±0,40
10-DOXYL	66,28±0,14	65,03±0,080 **
12-DOXYL	64,92±0,08	64,81±0,24
16-DOXYL	65,25±0,21	64,81±0,24

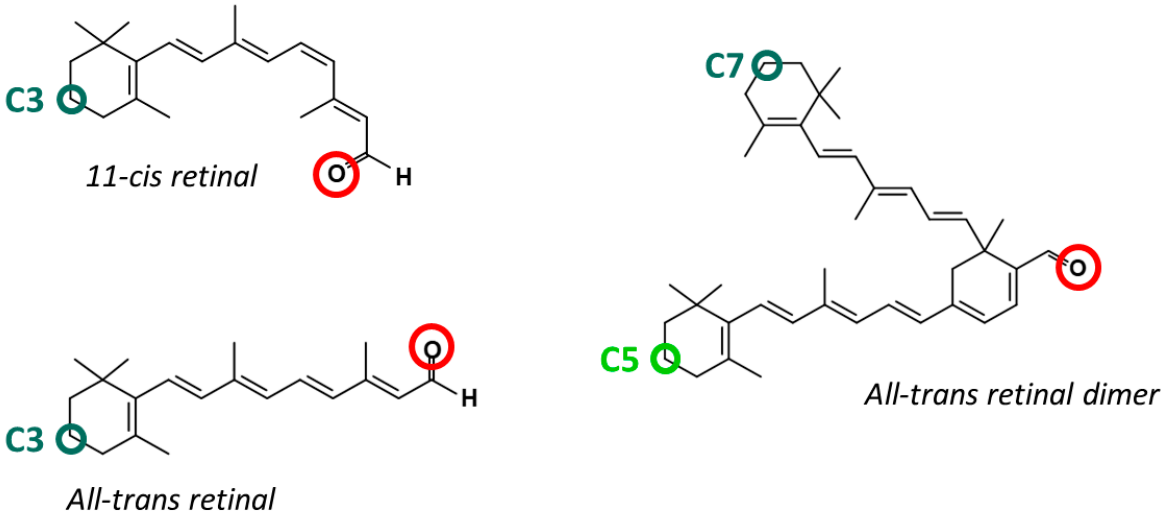

Figure S3. Structures of retinoids, location of which has been studied using MD in POPC model membrane, with selected atoms marked. Movement of marked atoms within POPC membrane has been tracked during simulations. Figures S4-S6 (see below) present Z-coordinate plots of these atoms in 11cRAL, AtRAL and AtRAL dimer molecules placed outside and inside POPC membrane.

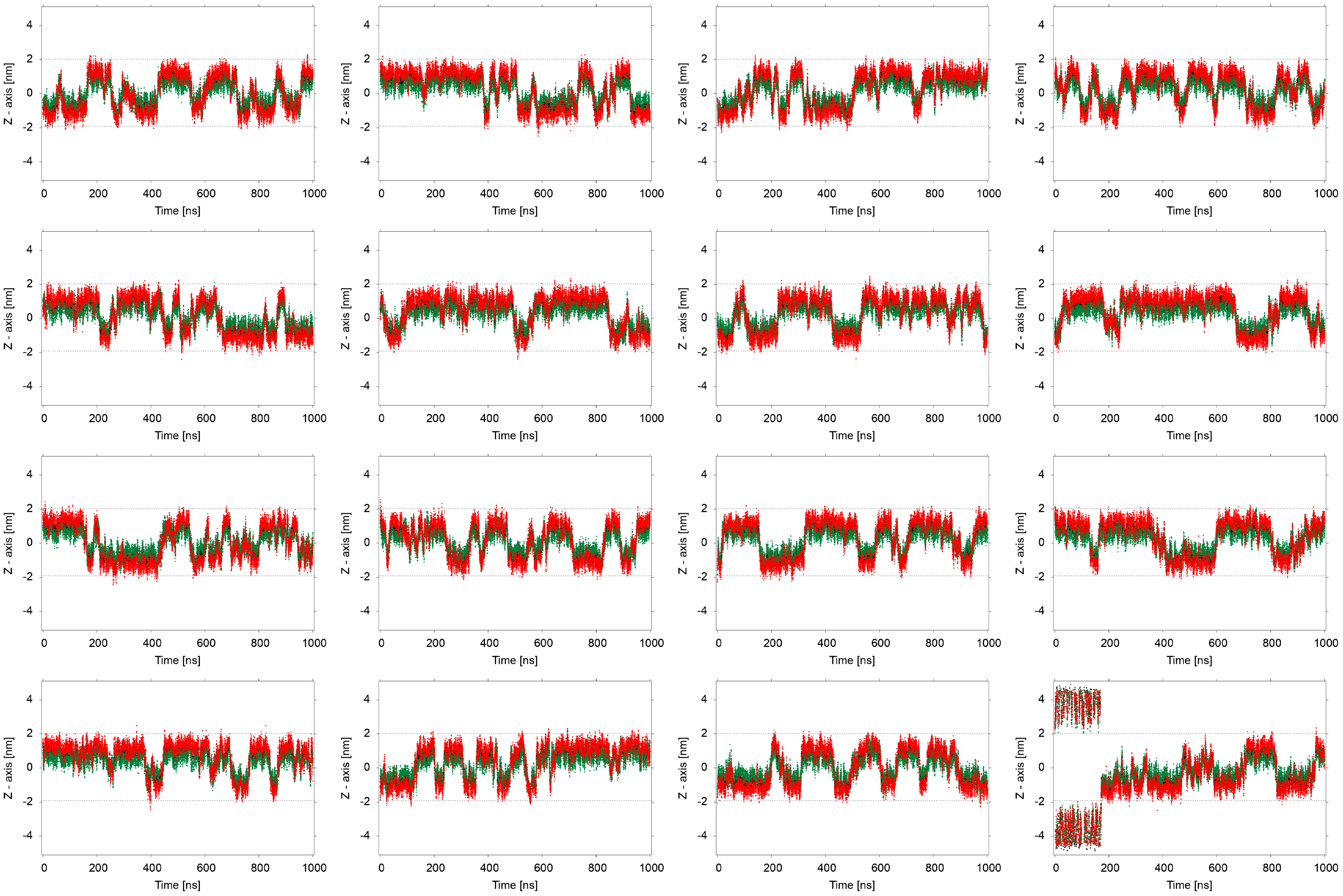
Z - axis [nm]
Time [ns]

Figure S4 *Z*–coordinate plots for the centre of mass (black line), oxygen (red) and carbon C3 (dark green) atoms of 16 molecules of 11-*cis* retinal. Dashed grey lines indicate the mean (from the entire simulation time) position of the P (phosphorus) atom of lipids.

At the starting point of simulation eight 11cRAL molecules were placed in the water phase near the interphase, parallel to the membrane surface. The other eight were located inside the bilayer parallelly to the POPC molecules, four of them with the carbonyl group directed to the water phase and four molecules with these groups directed to the hydrophobic bilayer core. Retinoid molecules placed outside the membrane entered the membrane immediately and located between POPC molecules, while retinoids placed inside the membrane with carbonyl group directed toward the membrane hydrophobic core, very quickly underwent reorientation placing the carbonyl group close to the surface of the membrane.

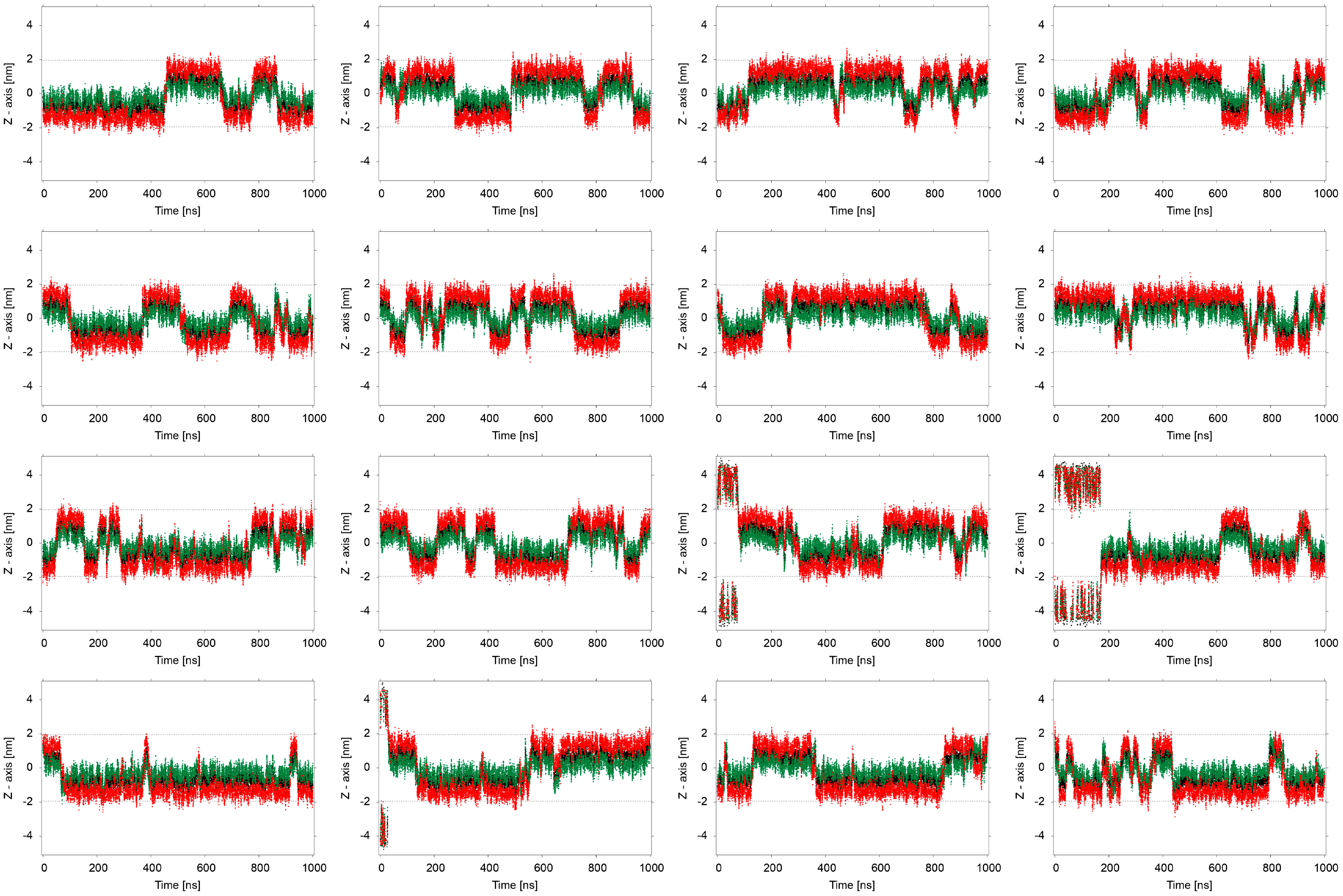
Z - axis [nm]
Time [ns]

Figure S5 Z–coordinate plots for the center of mass (black line), oxygen (red) and carbon C3 (dark green) atoms of 16 molecules of all-trans retinal. Dashed grey lines indicate the mean (from the entire simulation time) position of the P (phosphorus) atom of lipids.

At the starting point of simulation eight AtRAL molecules were placed in the water phase near the interphase, parallel to the membrane surface. The other eight were located inside the bilayer parallelly to the POPC molecules, four of them with the carbonyl group directed to the water phase and four molecules with these groups directed to the hydrophobic bilayer core. Retinoid molecules placed outside the membrane entered the membrane immediately and located between POPC molecules, while retinoids placed inside the membrane with carbonyl group directed toward the membrane hydrophobic core, very quickly underwent reorientation placing the carbonyl group close to the surface of the membrane.

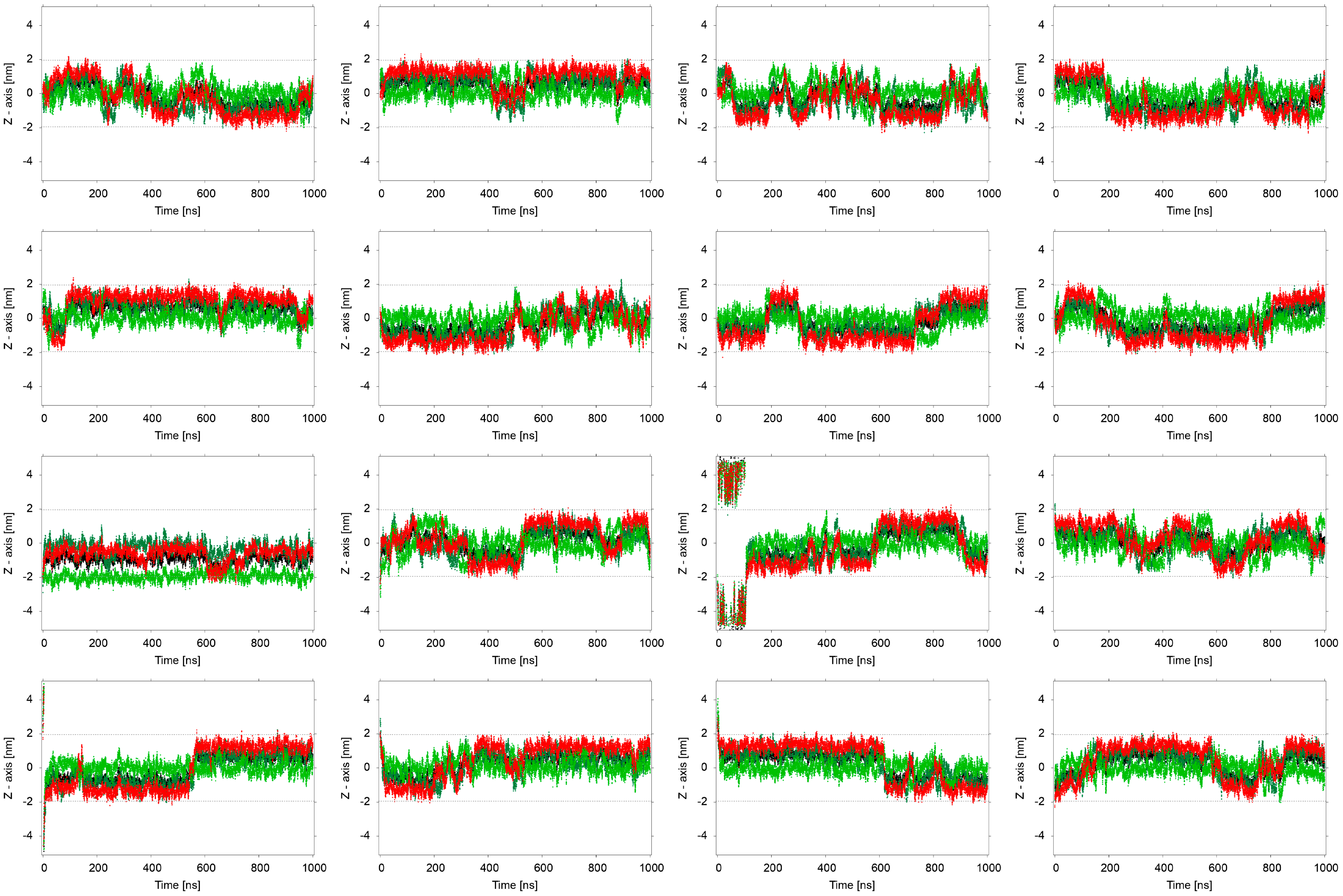
Z - axis [nm]
4
2
0
-2
-4
0
200
400
600
800
1000
Time [ns]

Figure S6 Z–coordinate plots for the center of mass (black line), oxygen (red), C5 (green) and C7 (dark green) carbon atoms of 16 molecules of AtRAL dimer. Dashed grey lines indicate the mean (from the entire simulation time) position of the P (phosphorus) atom of lipids.

At the starting point of simulation eight AtRAL dimer molecules were placed in the water phase near the interphase, parallel to the membrane surface. The other eight were located inside the bilayer parallelly to the POPC molecules, four of them with the carbonyl group directed to the water phase and four molecules with these groups directed to the hydrophobic bilayer core. AtRAL dimer molecules placed outside the membrane entered the membrane immediately and located between POPC molecules, while retinoids placed inside the membrane with carbonyl group directed toward the membrane hydrophobic core, very quickly underwent reorientation placing the carbonyl group close to the surface of the membrane.
